# The Question Concerning the Sensibility, Intelligence, and Instinctive Actions of Insects

**Published:** 1838-04

**Authors:** 


Art. III.—*The Question concerning the Sensibility, Intelligence, and Instinctive Actions of Insects.* By David Badham, m.d.; one of the Radcliffe Travelling Fellows of the University of Oxford, &c.—*Paris*, 1837. 8vo. pp. 54.

The ingenious author of this pamphlet has put forward much acute, but (as it appears to us) somewhat sophistical reasoning in defence of a position which will probably startle most of our readers,—that the insect tribes are not endowed with sensibility, still less with anything approaching to intelligence. Our own views on this subject have been partly expressed in a former article, and remain unchanged by the perusal of this essay: it contains, however, much clever argument and lively criticism upon the opinion of others, which will amuse, at least, if it does not convince. That a large proportion of the actions of the articulated tribes result from the stimulus of external *impressions* only, or, in the language of Dr. M. Hall, are *excito-motor*, we have no doubt; but this is only saying that they are purely *instinctive,* according to the definition we have given of the latter term. Dr. B.'s great error consists, as it appears to us, in maintaining that *sensations* can only act through the intellect and will: now, we have already endeavoured to show that this is by no means the case, and that there is a class of actions (of which the purely *emotional* movements may be taken as a specimen,) as necessarily connected with particular sensations as others are upon impressions only. We see no reason to believe that the *mental* emotion, passion, or propensity always intervenes, in the lower animals, between the sensation and the action: and, in fact, we are disposed to regard it as rather designed to awaken the reasoning faculties, and to exist therefore in proportion to them. But we feel no doubt that insects possess the senses of sight, hearing, &c. in great perfection, and that these senses minister to their instinctive actions. Nor can we refuse to them some degree of intelligence, which, we think, is proved by the power of adaptation to novel circumstances evidently possessed by many, and still more by the educability which some manifest. We have had the opportunity of noticing the last in the case of bees, whose *memory* for the entrance to a hive beneath a window, to which there were many similar, evidently improved during the first few days of their being placed in their new situation. We have an additional argument for the existence of sensation in insects in the fact that impressions in the special organs of the Vertebrata do not appear to operate like those which act through the organs of common sensation.

